# Oral tolerance induction protocols for treatment: an overview

**DOI:** 10.1186/2045-7022-1-S1-S78

**Published:** 2011-08-12

**Authors:** Wesley Burks

**Affiliations:** 1 Duke University Medical Center, Pediatric Allergy and Immunology, Durham, USA

## 

Food allergy is a major health concern in industrialized countries, affecting approximately 3.9% of children. Peanut allergy is one of the most common forms of food allergy, with approximately 3 million Americans reporting allergy to peanuts or tree nuts, and the prevalence appears to be increasing. Peanut and tree nut allergy account for the vast majority of life-threatening or fatal allergic reactions to foods. In addition, peanut allergy is often life-long, with only 20% of children outgrowing their allergy. Current treatment options are limited to strict peanut avoidance and ready access to epinephrine. Challenges for patients and families with food allergy are considerable, and accidental ingestion is common. Anxiety impairs social functioning and quality of life in food-allergic individuals, who report poorer health-related quality of life than those with diabetes mellitus. These findings underscore the need for active treatment strategies. Animal and human studies have examined potential therapies for peanut allergy, including allergen-nonspecific and allergen-specific modalities; however, recent meta-analyses highlight the shortage of controlled studies in the field. TNX-901, a humanized monoclonal antibody that binds IgE and prevents its binding to the high-affinity IgE receptor on mast cells and basophils, was found to increase the threshold of peanut protein inducing symptoms in peanut-allergic individuals from less than one peanut (178 mg) to almost twelve peanuts (2805 mg). However, this effect was seen only at the highest dose tested, and the prohibitive cost of monoclonal antibody treatment may limit this approach. A combination of traditional Chinese herbal medications, food allergy herbal formula 2 (FAHF-2), has shown promise in eliminating anaphylaxis to peanut in murine and phase I studies. Allergen immunotherapy, an allergen-specific treatment, refers to the administration of increasing amounts of an allergen to individuals with IgE-mediated allergy in order to induce immunologic changes which diminish the allergic response to the substance on subsequent encounters. Traditional subcutaneous immunotherapy with aqueous peanut extract was attempted but had an unacceptably high rate of systemic reactions, despite favorable challenge outcomes.

In pilot studies, our group has shown that open-label peanut oral immunotherapy (OIT) was relatively safe when performed in a supervised medical setting by trained personnel and was associated with clinical desensitization for the majority of subjects who completed more than eight months of treatment. We use the term desensitization to signify a change in threshold of ingested food antigen needed to cause allergic symptoms; this state is dependent on regular exposure to the allergen. In contrast, tolerance refers to the induction of long-term immunologic changes associated with the ability to ingest a food without symptoms and without ongoing therapy.

In order to establish the safety and efficacy of peanut OIT as an allergen-specific therapy for peanut allergy, we conducted the first randomized, double-blind, placebo-controlled study of OIT in children with peanut allergy. Our goals were to evaluate the ability of peanut OIT to safely raise the threshold of ingested food antigen when compared with placebo and to perform immunologic studies to investigate the underlying mechanisms associated with clinical efficacy. The primary endpoint was to determine if, after one year of treatment, subjects receiving peanut OIT would be able to ingest a greater amount of peanut protein at food challenge than those receiving placebo.

